# Supplementary material for: Supramolecular Chromatographic Separation of C60 and C70 Fullerenes: Flash Column Chromatography vs. High Pressure Liquid Chromatography
Source: Int J Mol Sci. 2021 May 27;22(11):5726. doi: 10.3390/ijms22115726 (PMC8198677; doi:10.3390/ijms22115726)
Supplement: Supplementary file 1 [file ijms-22-05726-s001.zip › ijms-1217990-supplementary.pdf]

Supporting Information

Supramolecular chromatographic separation of C_{60} and C_{70} fullerenes: flash column chromatography versus high pressure liquid chromatography

Subbareddy Mekapothula[1], A. D. Dinga Wonanke[1], Matthew A. Addicoat[1], David J. Boocock[2], John D. Wallis[1] and Gareth W. V. Cave[1]*

[1]School of Science and Technology, Nottingham Trent University, Clifton Lane, Nottingham NG11 8NS, UK.
[2]The John van Geest Cancer Research Centre, Nottingham Trent University, Clifton Lane, Nottingham, NG11 8NS, UK.

Contents

1. Characterization of *C*-bromo-butylpyrogallol[4]arene **2 ERROR! BOOKMARK NOT DEFINED.**

2. Raw computational data ____3

3. Binding energies and modes for c60⊂rp-c18 and c70⊂rp-c18 ______________________3

1. Characterization of C-bromo-butylpyrogallol[4]arene **2**

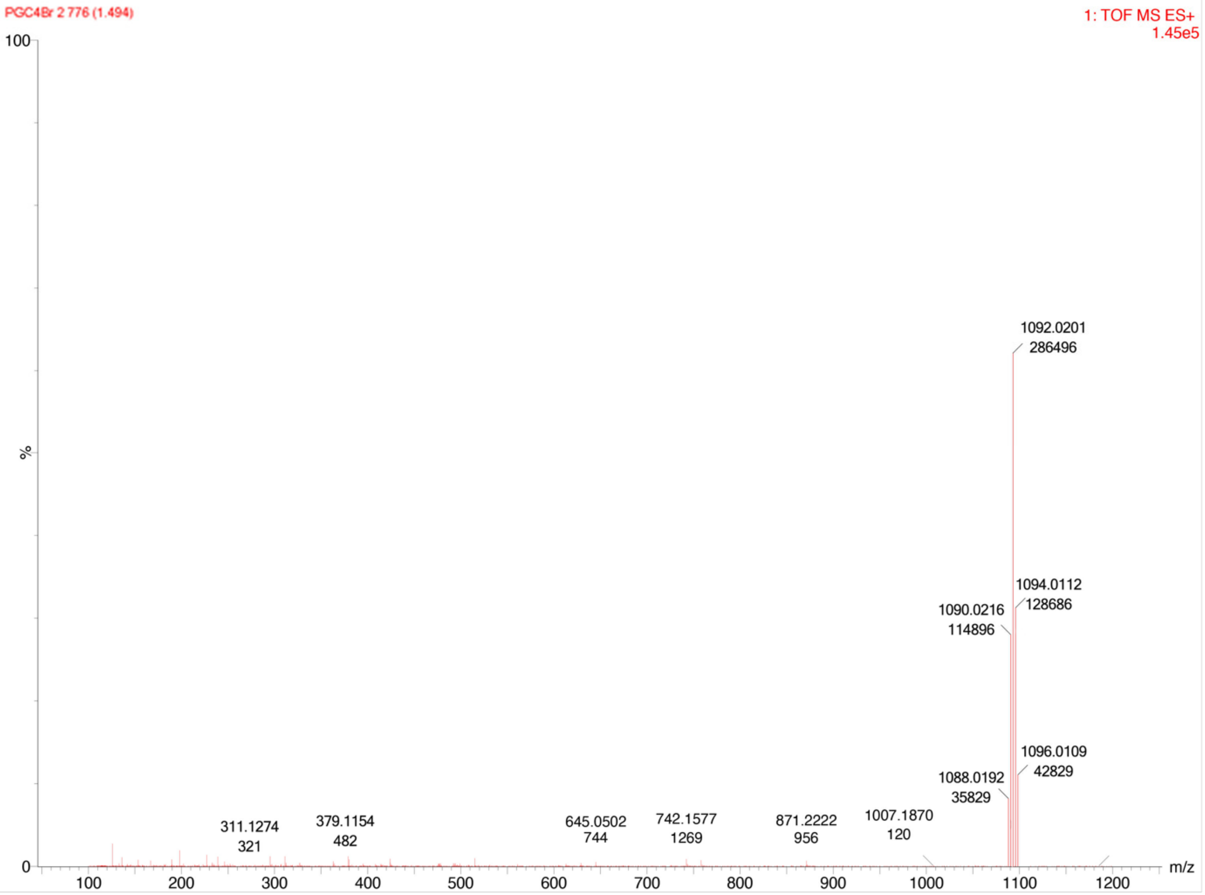

Figure S1. Mass Spectrum of *C*-bromo-butylpyrogallol[4]arene **2**. ESI-MS *m/z* calculated for $C_{44}H_{52}{}^{79}Br_4O_{12}$ $[M]^+$ was 1088.01, found for $[M]^+$ to $[M+8]^+$ were 1088.0192,1090.0216, 1092.0201, 1094.0112 and 1096.0109.

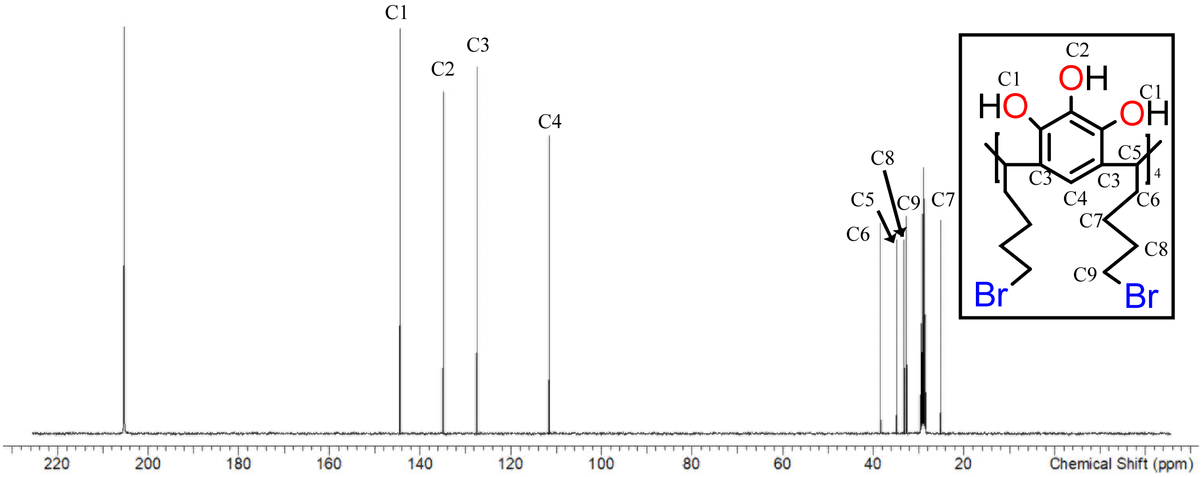

Figure S2. ^{13}C NMR spectrum (101 MHz, d_6-Acetone, room temperature) of *C*-bromo butylpyrogallol[4]arene **2**.

2. Computational data

All the structures, long range interactions and energies of these 20 energy minima can be freely accessed at http://doi.org/10.5281/zenodo.4147306.

3. Binding energies and modes for C_{60}⊂RP-C_{18} and C_{70}⊂RP-C_{18}

To gauge the interactions between RP-C_{18} and the fullerenes, a host-guest interaction study was performed to compute the binding energies of C_{60}⊂RP-C_{18} and C_{70}⊂RP-C_{18} motifs. This was done using two conformers of RP-C_{18}, a straight chain and a curved conformer as input structures. These computations were performed for both the gas and solvent phase using the same level of theory as stated in the main text for the computation of the host-guest interactions of fullerene⊂*C*-butylpyrogallol[4]arene complexes. The computed gas and solvent phase Gibbs free binding energies (ΔGBE) and electronic energies are presented Table S1. The detailed energetic parameters are presented in Table S2 and S3 for the gas and solvent phases respectively.

It can be observed from Table S1 that the interaction energies of C_{70}⊂RP-C_{18} are much lower (nearer to negative) in comparison to C_{60}⊂RP-C_{18} both in the gas and solvent phase. This shows a stronger binding affinity of C_{70} to RP-C_{18} in comparison to C_{60}, consequently resulting in a preferential selective separation of C_{70} over C_{60}.

However, the small positive Gibbs free binding energies for these interactions indicate that the interactions between RP-C_{18} and the fullerenes are significantly weak (thermodynamically unfovorable) and consequently the RP-C_{18} stationary phase is less favourable for the separation of fullerenes in comparison to *C*-butylpyrogallol[4]arene.

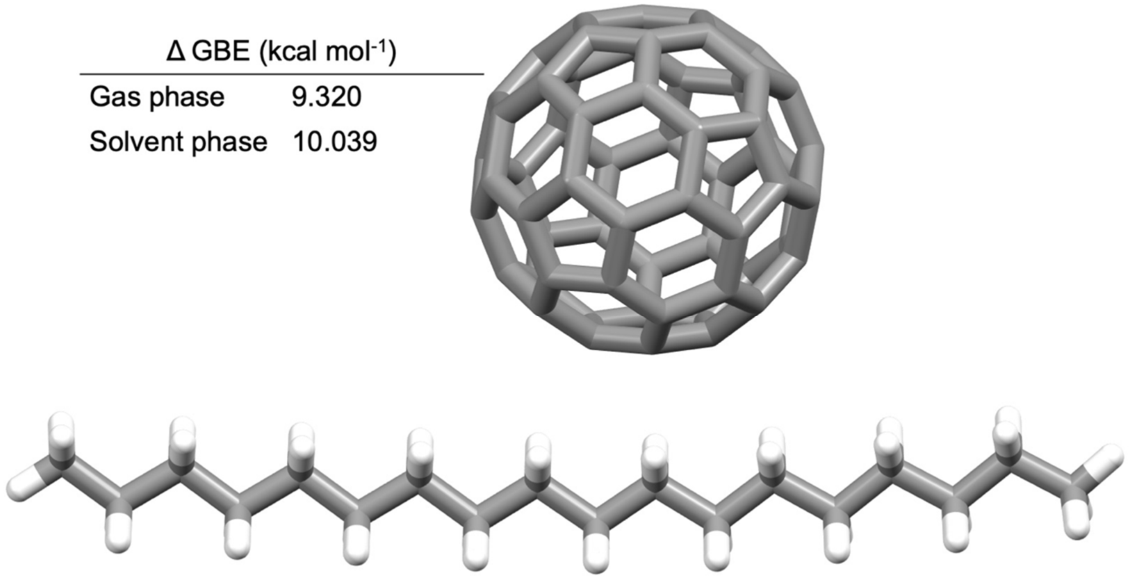

a

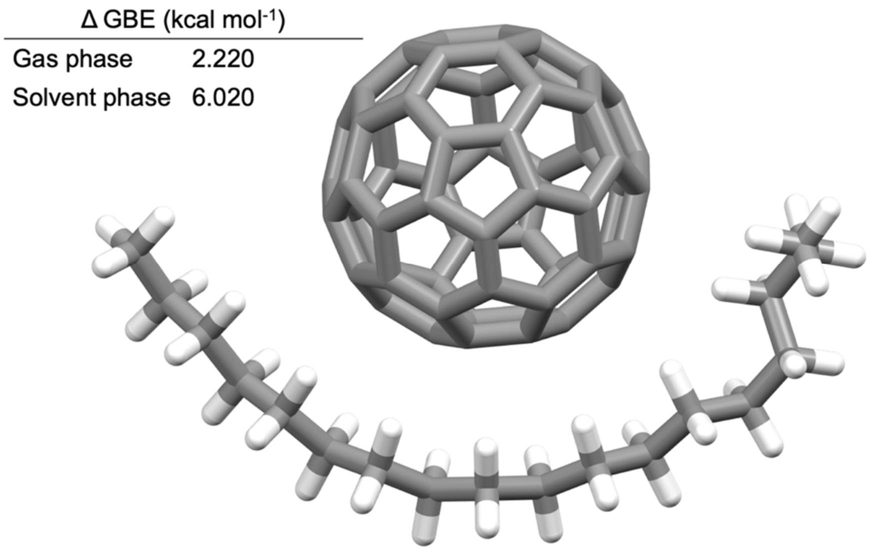

b

Figure S3: Illustration of the calculated gas and solvent phase binding energies and modes of $C_{60}\subset$RP-C_{18} interaction: a) represents the binding mode between the straight chain conformer of RP-C_{18} and C_{60}, and b) represents the binding mode between the curved conformer of RP-C_{18} and C_{60}.

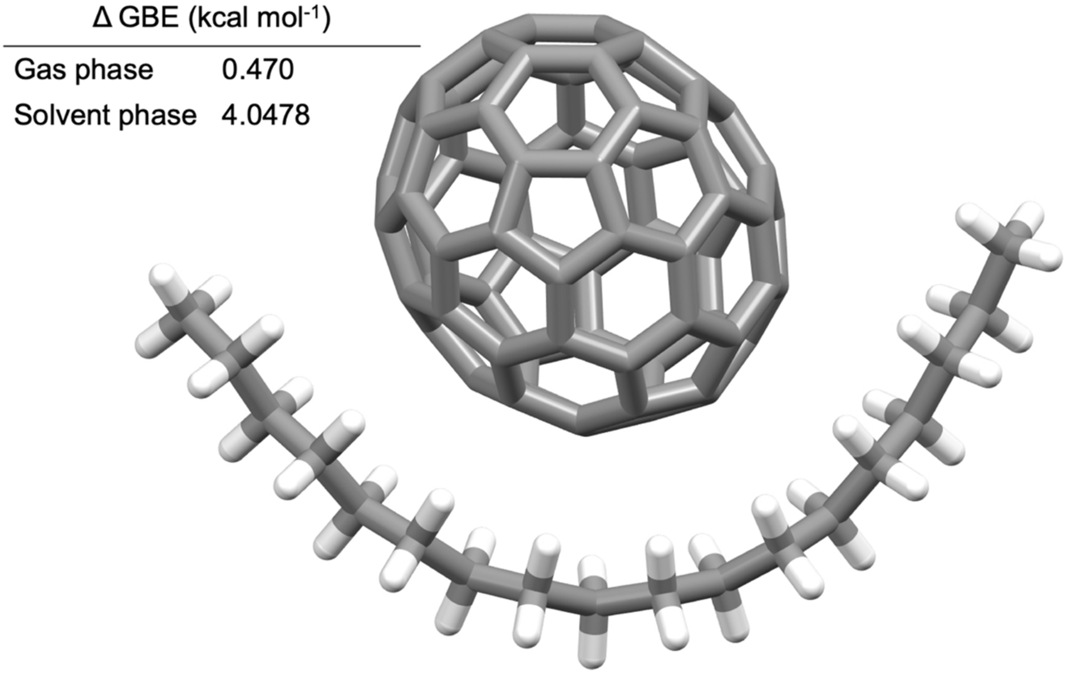

a

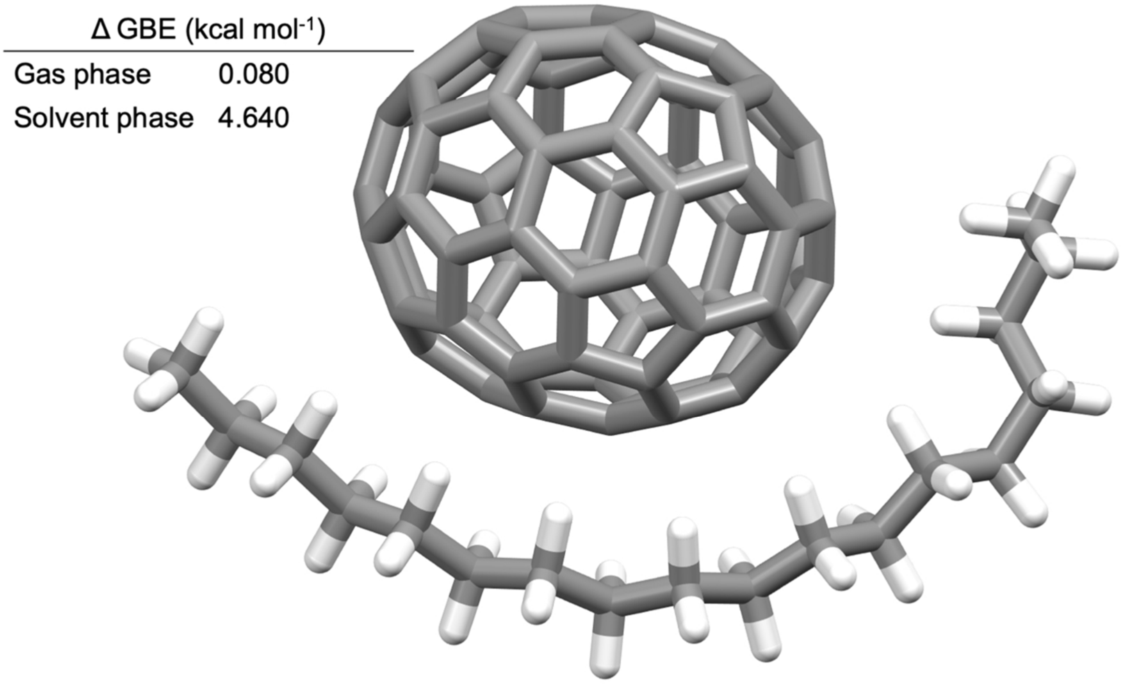

b

Figure S4: Illustration of the calculated gas and solvent phase binding energies and modes of $C_{70} \subset$RP-C_{18}. a and b represent the binding mode of two curved conformers of RP-C_{18} with C_{70}.

Table S1: Change in electronic energy and Gibbs free binding energy of fullerene⊏RP-C_{18} motifs.

Systems	Gas phase (kcal mol^{-1})		Solvent phase (kcal mol^{-1})	
	ΔE	ΔGBE	ΔE	ΔGBE
C_{60}⊏RP-C_{18}-a	-0.0153	9.320	-9.478	10.039
C_{60}⊏RP-C_{18}-b	-0.028	2.220	-12.987	6.020
C_{70}⊏RP-C_{18}-a	-0.0311	0.470	-14.315	4.0478
C_{70}⊏RP-C_{18}-b	-0.0307	0.080	-14.301	4.640

E is the electronic change of the system, in practice is the final optimised energy the system. *ΔE* is the change in electronic energy between the complex and the isolated systems.

$$\Delta E = E_{fullerene \sqsubset RP-C_{18}} - (E_{fullerene} - E_{RP-C_{18}})$$

$E_{fullerene \sqsubset RP-C18}$ is the electronic energy of the fullerene⊏RP-C_{18} motif. $E_{fullerene}$ is the electronic energy of the isolated fullerene and E_{RP-C18} is the electronic energy of the isolated RP-C_{18}.

GBE is the Gibbs free energy of the system. This was computed as follows:

$$GBE = E + H - TS$$

H is the thermal correction to the enthalpy of the system. *S* is the thermal correction to entropy. *T* is the temperature of the system which is equal to 298.15 K.

$$\Delta GBE - GBE_{fullerene \sqsubset RP-C_{18}} - (GBE_{fullerene} - GBE_{RP-C_{18}})$$

$GBE_{fullerene \sqsubset RP-C18}$ is the Gibbs free energy of the fullerene⊏RP-C_{18} motif. $GBE_{fullerene}$ is the Gibbs free energy of the isolated fullerene and G_{RP-C18} is the Gibbs free energy of the isolated RP-C_{18}.

Table S2: Gas Phase UFF DFTB Mio-1-1 Contributions the Gibbs Free Energy (Hartree)

Systems	Electronic energies	RT	-TS	Internal energies	Enthalpies	Gibbs Free energies
C_{60}	-102.6746482	0.000944186	-0.064338271	-102.2522524	-102.2513083	-102.3156465
C_{70}	-119.8820304	0.000944186	-0.070701247	-119.387666	-119.3867218	-119.4574231
RP-C_{18}	-45.10614778	0.000944186	-0.079772362	-44.55891042	-44.55796623	-44.63773859
$C_{60}\subset$RP-C_{18}-a	-147.7961452	0.000944186	-0.111769899	-146.8277065	-146.8267623	-146.9385322
$C_{60}\subset$RP-C_{18}-b	-147.8088012	0.000944186	-0.111966711	-146.8388275	-146.8378833	-146.9498501
$C_{70}\subset$RP-C_{18}-a	-165.0192592	0.000944186	-0.117773367	-163.9775864	-163.9766423	-164.0944156
$C_{70}\subset$RP-C_{18}-b	-165.0188829	0.000944186	-0.118983495	-163.976999	-163.9760548	-164.0950383

Table S3: Solvent Phase UFF DFTB Mio-1-1 Contributions the Gibbs Free Energy (Hartree)

Systems	Electronic energies	RT	-TS	Internal energies	Enthalpies	SASA Free Energy	Gibbs Free energies
C_{60}	-102.7139323	0.000944186	-0.064366	-102.291834	-102.2908898	-0.03929056	-102.3552558
C_{70}	-119.9269967	0.000944186	-0.07072882	-119.4329698	-119.4320256	-0.04497303	-119.5027544
RP-C_{18}	-45.13065122	0.000944186	-0.07933919	-44.58411324	-44.58316905	-0.02453648	-44.66250824
$C_{60}\subset$RP-C_{18}-a	-147.8596879	0.000944186	-0.10955973	-146.8931503	-146.8922061	-0.06372944	-147.0017658
$C_{60}\subset$RP-C_{18}-b	-147.8652793	0.000944186	-0.11301986	-146.8960955	-146.8951513	-0.05785244	-147.0081712
$C_{70}\subset$RP-C_{18}-a	-165.0804601	0.000944186	-0.12000952	-164.0397467	-164.0388025	-0.06170926	-164.158812
$C_{70}\subset$RP-C_{18}-b	-165.080438	0.000944186	-0.1194558	-164.0393571	-164.038413	-0.06209017	-164.1578688
